# Quantifying the speed-accuracy trade-off of large language models on oral and maxillofacial surgery multiple-choice questions

**DOI:** 10.1038/s41598-025-27256-7

**Published:** 2025-11-19

**Authors:** Viet Anh Nguyen, Thi Bich Ngoc Ha, Minh Ngoc Tran, Ngo The Minh Pham, Thuy Linh Nguyen, Thi Quynh Trang Vuong

**Affiliations:** 1 Faculty of Dentistry, Phenikaa University, Hanoi, Vietnam; 2 Private Practice, Viet Anh Orthodontic Clinic, Hanoi, Vietnam

**Keywords:** Artificial intelligence, Large language model, Oral and maxillofacial surgery, Multiple-choice examination, Diagnostic accuracy, Response latency, Dentistry, Oral medicine, Oral pathology

## Abstract

Large language models (LLMs) such as GPT-4o, Copilot and Gemini are entering dental curricula, yet their suitability for real-time decision support remains unclear because most evaluations report accuracy alone. This prospective in silico diagnostic-accuracy study benchmarked six engines—GPT-4o, OpenAI o3, Copilot-Quick, Copilot-Deep, Gemini-Flash and Gemini-Pro—against 1766 single-best-answer multiple-choice questions from a contemporary oral and maxillofacial surgery (OMFS) board-review text. Textbook keys served as the reference standard. Overall and domain-level accuracy, intra-model answer consistency and per-batch response latency were recorded; χ² tests compared accuracies and Kruskal–Wallis with multiplicity-adjusted Mann–Whitney U tests compared response times. Accuracy differed significantly across engines (χ² = 97.31, *p* < 0.001), ranging from 77.9% for Copilot-Quick to 88.3% for Gemini-Pro. Reasoning-optimised variants (o3, Copilot-Deep, Gemini-Pro) exceeded their speed-tuned counterparts by 3.8–6.2% points, with the largest gains in trauma, craniofacial deformity and orthognathic surgery domains. These improvements incurred a marked latency penalty: median response times of 2.1–3.1 s versus 0.1–0.2 s for the faster engines. Each additional 3–6 correct answers per 100 items therefore required roughly 2–3 s of extra processing. Items unanswered by all models clustered around rare numeric facts and negatively worded stems. Reasoning-optimised LLMs deliver clinically meaningful accuracy gains on OMFS board questions, but educators and clinicians must weigh this benefit against slower output and maintain expert oversight to mitigate residual knowledge gaps.

**Supplementary Information:**

The online version contains supplementary material available at 10.1038/s41598-025-27256-7.

## Introduction

Large-language models (LLMs) such as GPT-4o, Claude-3, Gemini-Pro and Microsoft Copilot have rapidly permeated biomedical education. In dentistry, they are already being used to draft teaching material, simulate viva voces and grade multiple-choice examinations, prompting calls for systematic evaluation of their reliability and clinical relevance^1^. A recent systematic review identified 46 peer-reviewed studies of LLMs in dentistry but noted that most analyses stop at answer correctness, leaving practical metrics such as response latency unexplored^2^.

Within oral-maxillofacial surgery (OMFS), GPT-4 has demonstrated 62% overall accuracy on specialty-board questions, with performance varying from 93% in pharmacology to 38% in orthognathic surgery^3^. In general dentistry assessments, ChatGPT-4 consistently outperforms both ChatGPT-3.5 and traditional study resources; for example, it scored 76.9% on the Integrated National Board Dental Examination compared with 61.3% for its predecessor^4^. Parallel work in oral disease diagnostics reported an 86.1% correct-response rate for ChatGPT-4o, underlining the model’s potential when domain prompts are carefully engineered^5^.

Collectively, these investigations confirm that LLMs can reach or surpass human pass-marks across diverse dental sub-disciplines. Yet all of them report a single metric, accuracy, and none quantify the time the model needs to deliver each answer, a variable that strongly determines their feasibility for chair-side decision support or real-time teaching. The absence of speed data is especially conspicuous in oral-maxillofacial medicine, where clinicians often require on-the-spot clarification of complex pharmacotherapeutic or pathology queries.

To address this gap, the present study concurrently measures answer correctness and per-question latency for two configurations of the same LLM, one tuned for maximal accuracy and another tuned for minimal response time. We focus on a validated pool of oral-maxillofacial medicine multiple-choice questions so that results speak directly to day-to-day clinical education. We hypothesised that the accuracy-oriented configuration would achieve a significantly higher correct-response rate than the latency-oriented configuration, but only at the cost of longer median response times, thereby revealing a quantifiable inverse relationship between speed and accuracy in oral-maxillofacial medicine queries. Because response latency scales with generated tokens and scoring rules differ by item type, we restricted our evaluation a priori to single-best-answer multiple-choice questions (MCQs) to isolate a clean speed–accuracy trade-off. Multi-select and open-ended formats require distinct scoring and power considerations and are reserved for a dedicated follow-up study.

## Materials and methods

### Study design

We conducted a prospective, in-silico diagnostic-accuracy study that compared the performance of a large language model (LLM) with an expert answer key on MCQs in oral and maxillofacial surgery. The study employed a cross-sectional design, adhered to the Strengthening the Reporting of Observational Studies in Epidemiology (STROBE) checklist. The study relied solely on publicly available, non-identifiable educational material and involved no human participants, institutional review board approval was therefore not necessary. All methods were carried out in accordance with relevant guidelines and regulations.

Based on the effect size (w = 0.10) reported by Nguyen et al.^6^, an a priori sample size calculation for a χ² test comparing six groups at α = 0.01 and 99% power indicated that approximately 560 observations per group were necessary.

### Question source and taxonomy

**Fig. 1:**
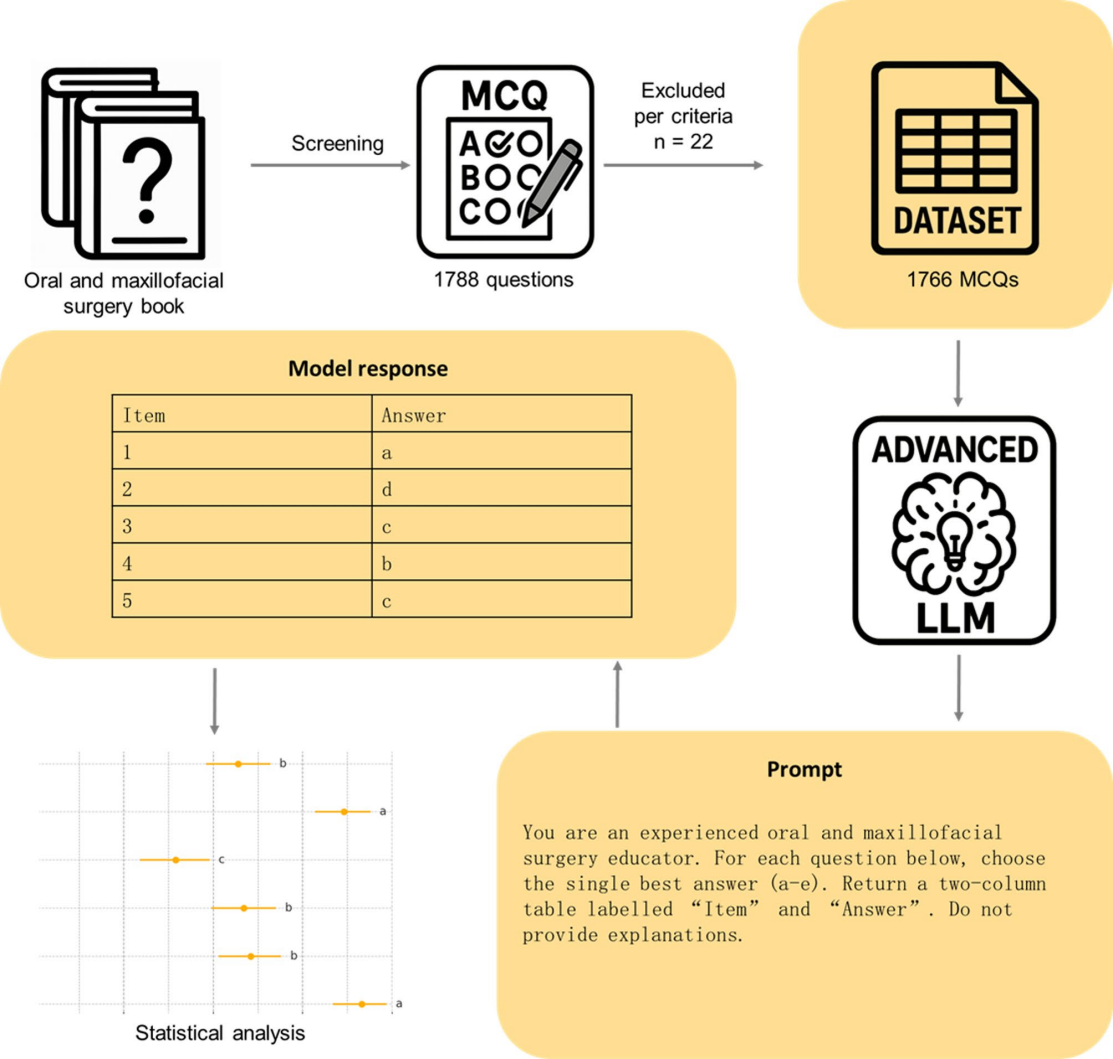
Study workflow diagram. LLM, large-language models; MCQ, multiple-choice questions.

A single commercially available 2024 board-review text in oral and maxillofacial surgery was used as the exclusive data source because its recently updated edition offers a particularly large, curriculum-aligned pool of examination questions, ensuring both contemporary relevance and sufficient item numbers for robust analysis^7^. After screening 1,788 items, which comprised open-ended, true/false, multi-select, and single-best-answer formats, and removing duplicates as well as vignette-dependent stems, 1766 unique single-best-answer MCQs remained (Fig. 1). Each item was assigned to one of twelve domains defined by the textbook index, including anatomy, anaesthesia and pharmacology, craniofacial deformity, cosmetic procedures, dental implants, dentoalveolar surgery, orthognathic surgery and sleep apnoea, infection and pathology, medicine, reconstructive and soft-tissue surgery, temporomandibular joint, and trauma. Non-single-best-answer items were excluded because their heterogeneous scoring (all-or-none versus partial credit) and variable option structures are not commensurable with single-best-answer accuracy and would confound latency estimates.

### LLM configuration and prompting

Three groups of LLM were benchmarked to compare next-generation deep-reasoning engines with earlier systems. Group 1 included OpenAI’s o3, chosen for its advanced reasoning capability, and GPT-4o, which served as the baseline control for performance comparisons^8^. Group 2 used Microsoft Copilot, built on an o3-mini architecture, and was assessed in both quick-response and deep-reasoning formats to capture its dual latency profiles. Group 3 included Google’s Gemini 2.5 Flash, optimised for low-latency output, and Gemini 2.5 Pro, designed for more deliberate reasoning on complex tasks. These engine groups were selected because recent citation and usage surveys rank them among the most common platforms in dental-education research over the previous 12 months^6^.

Inference was executed in June 2025 through each vendor’s official application-programming interface (API) using default settings (temperature = 1). Questions were processed in mini-batches of ten, in which every request contained the full stem and answer options for ten MCQs, excluding the keyed responses and domain labels. The accompanying system prompt read:

“You are an experienced oral and maxillofacial surgery educator. For each question below, choose the single best answer (a-e). Return a two-column table labelled “Item” and “Answer”. Do not provide explanations.”

Suppressing explanations served two purposes. First, it eliminated variability in the number of output tokens, because inference latency in LLMs scales roughly linearly with generated tokens. Second, it allowed response time to reflect the model’s internal reasoning speed rather than its verbosity, thereby enabling fair comparisons across engines. Each batch was submitted in a fresh session to eliminate cross-item memory. Per-batch response times were recorded to enable direct speed comparisons among the six models.

### Outcome measures

Textbook answer keys served as the reference standard. Two board-certified oral and maxillofacial surgeons independently audited a random 10% sample of items against the source text; inter-rater agreement was 100%, so the remaining keys were accepted without further review.

To evaluate intra-model stability, a separate random 10% of the question bank was resubmitted to each LLM in an additional batch, and consistency was defined as the proportion of identical answers across the two passes. The primary outcome was overall accuracy, expressed as the proportion of items answered correctly. Secondary outcomes were domain-specific accuracy, intra-model consistency, and response latency.

### Statistical analysis

Exact 95% confidence intervals (CIs) for proportions were calculated with the Clopper–Pearson method. Overall and domain-level differences in accuracy across the six LLMs were assessed with χ² tests, and significant omnibus results were explored by pairwise comparisons adjusted with the Benjamini-Hochberg procedure. The same χ²-based framework was applied to inter-model answer-consistency rates. Response-time data were non-normally distributed (Shapiro–Wilk *P* < 0.05) and therefore analysed with the Kruskal–Wallis test, followed by pairwise Mann–Whitney U tests with Bonferroni correction. All statistics were performed in IBM SPSS Statistics, version 26.0 (IBM Corp., Armonk, NY, USA). Two-tailed *P* < 0.05 after adjustment was considered significant. A priori power calculations targeted χ² comparisons across six engines within an single-best-answer framework; incorporating a small, imbalanced multi-select subset post hoc would be under-powered after multiple-comparison control and thus was not attempted.

## Results

### Overall accuracy

**Fig. 2:**
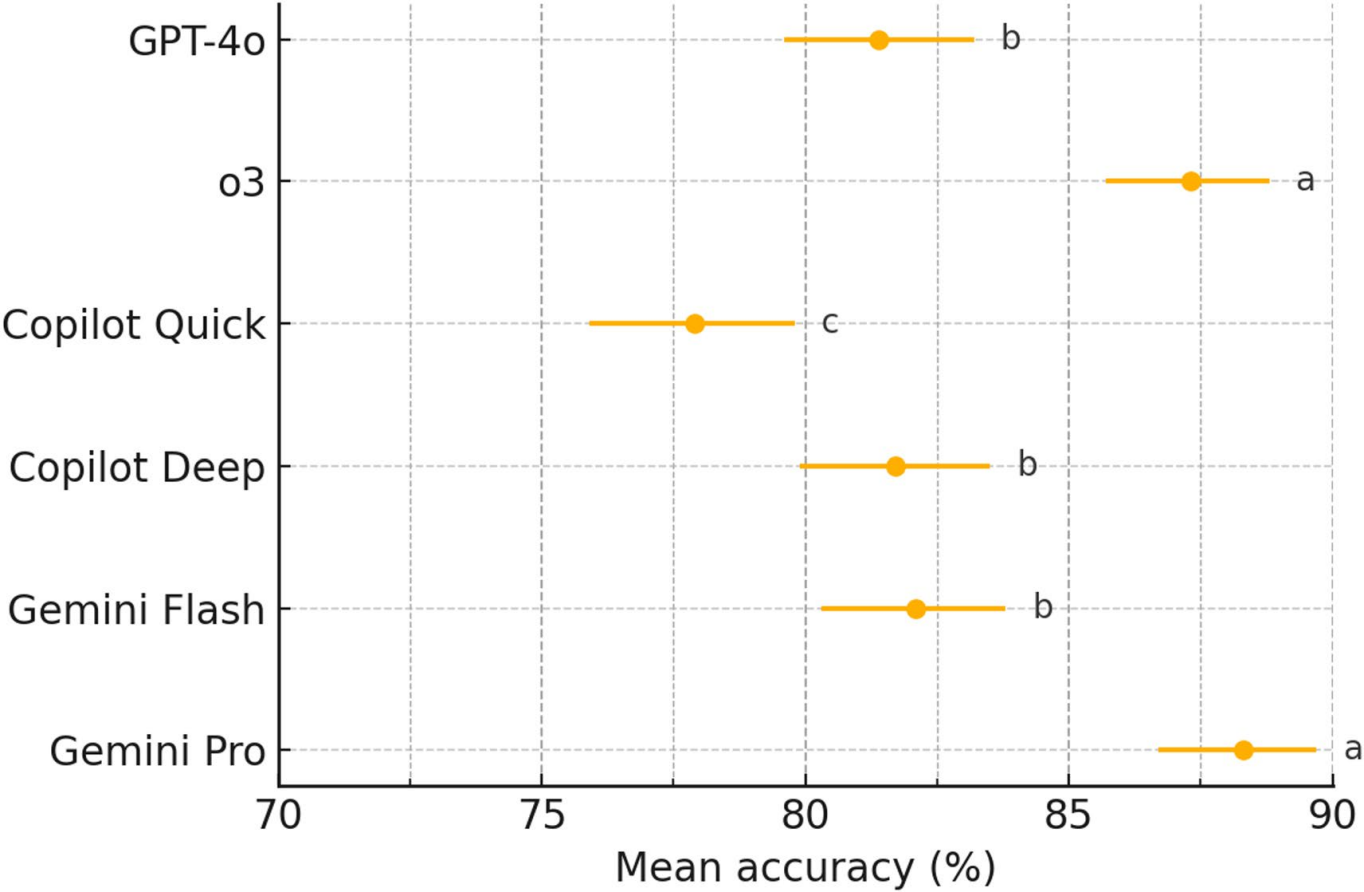
Forest plot summarising accuracy for each language model, with 95% confidence intervals. Labels that carry the same lowercase letter (**a-c**) do not differ after Benjamini–Hochberg–adjusted pairwise testing.

**Fig. 3:**
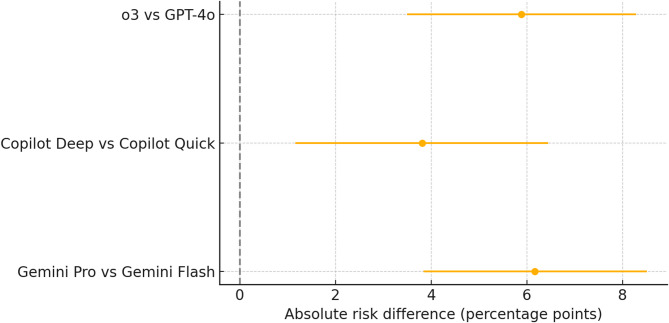
Forest plot displaying absolute risk-difference estimates (percentage points) for the three within-vendor comparisons, including o3 versus GPT-4o (OpenAI), Copilot-Deep versus Copilot-Quick, and Gemini-Pro versus Gemini-Flash.

Across the 1766 single-best-answer items, model accuracy ranged from 77.9% (95% CI 75.9–79.8) for Copilot-Quick to 88.3% (95% CI 86.7–89.7) for Gemini-Pro, with intermediate accuracies of 81.4% (95% CI 79.6–83.2) for GPT-4o, 81.7% (95% CI 79.9–83.5) for Copilot-Deep, 82.1% (95% CI 80.3–83.8) for Gemini-Flash, and 87.3% (95% CI 85.7–88.8) for o3. A global χ² test confirmed a significant performance gap among the six engines (χ² = 97.31, *P* < 0.001; Table 1). Benjamini–Hochberg-adjusted pairwise tests identified several significant differences (Fig. 2). Within-group comparisons showed that o3 outperformed GPT-4o by 5.89% points (95% CI 3.50–8.28; *P* < 0.001, Benjamini-Hochberg adjusted), Copilot-Deep exceeded Copilot-Quick by 3.81 points (95% CI 1.16–6.45; *P* = 0.007), and Gemini-Pro surpassed Gemini-Flash by 6.17 points (95% CI 3.84–8.51; *P* < 0.001; Fig. 3).

**Table 1:** Accuracy of six large-language models across oral and maxillofacial surgery domains. Each cell shows the item count (n) and accuracy expressed as a percentage with its 95% confidence interval. Pearson χ² tests with Benjamini-Hochberg correction were used to compare model performance within every domain.

Category	*n*	GPT − 4o	GPT-o3	Copilot Quick	Copilot Deep	Gemini Flash	Gemini Pro	*P* Value
Anatomy	99	82.8 74.5–89.3	97.0 92.1–99.1	84.8 76.8–90.9	80.8 72.2–87.6	86.9 79.2–92.4	91.9 85.3–96.1	0.017
Anaesthesia and pharmacology	209	88.0 83.1–91.9	93.8 89.9–6.5	86.1 81.0-90.3	87.6 82.6–91.5	86.6 81.5–90.7	90.0 85.3–93.5	0.196
Craniofacial deformity	96	89.6 82.3–94.5	91.7 84.9–96.0	82.3 73.8–88.9	90.6 83.6–95.3	93.8 87.6–97.3	97.9 93.599.6	0.019
Cosmetic procedures	142	82.4 75.5–88.0	90.1 84.4–94.2	73.9 66.3–80.6	79.6 72.4–85.6	85.9 79.5–90.9	89.4 83.6–93.7	0.008
Dental implants	91	84.6 76.2–90.9	86.8 78.7–92.6	84.6 76.2–90.9	83.5 74.9–90.0	82.4 73.6–89.2	87.9 80.1–93.4	0.914
Dentoalveolar surgery	112	67.0 57.9–75.2	77.7 69.3–84.6	66.1 57.0-74.3	74.1 65.5–81.5	69.6 60.7–77.6	76.8 68.4–83.9	0.269
Orthognathic surgery and sleep apnea	140	67.1 59.1–74.5	73.6 65.8–80.3	62.9 54.7–70.5	63.6 55.4–71.2	60.7 52.5–68.5	79.3 72.0-85.4	0.017
Infection and pathology	263	93.2 89.6–95.7	91.6 87.8–94.5	87.5 83.0–91.0	90.1 86.1–93.3	90.1 86.1–93.3	92.0 88.3–94.8	0.311
Medicine	215	86.0 80.9–90.2	91.2 86.8–94.4	85.1 79.9–89.4	91.6 87.4–94.8	87.9 83.0-91.8	93.5 89.6–96.2	0.054
Reconstructive and soft tissue surgery	90	73.3 63.6–81.6	80.0 70.9–87.2	66.3 56.1–75.5	74.4 64.8–82.6	73.3 63.6–81.6	83.3 74.6–89.9	0.196
Temporomandibular joint	99	78.8 70.0-85.9	85.9 78.0-91.7	71.7 62.3–79.9	74.7 65.6–82.5	75.8 66.7–83.4	82.8 74.5–89.3	0.196
Trauma	210	71.0 64.6–76.8	81.4 75.8–86.2	69.0 62.6–75.0	74.8 68.6–80.3	79.0 73.2–84.1	86.7 81.6–90.8	< 0.001
Total sample	1766	81.4 79.6–83.2	87.3 85.7–88.8	77.9 75.9–79.8	81.7 79.9–83.5	82.1 80.3–83.8	88.3 96.7–89.7	< 0.001

### Domain-level accuracy

Significant inter-model differences were confined to the anatomy (*P* = 0.017, Benjamini-Hochberg adjusted), craniofacial deformity (*P* = 0.019), cosmetic procedures (*P* = 0.008), Orthognathic surgery and sleep apnea (*P* = 0.017), and trauma (*P* < 0.001) domains; no meaningful disparities emerged in the remaining seven categories (*P* > 0.05). The highest single accuracy was observed in craniofacial deformity, where Gemini-Pro answered 97.9% correctly, whereas the lowest occurred in orthognathic surgery and sleep apnea, with Gemini-Flash scoring 60.7%.

### Answer consistency

Resubmitting a random 10% subset of items (*n* = 170 per model) revealed a significant overall difference in intra-model stability (χ² = 15.15, *P* = 0.010). Consistency rates were: GPT-4o 92.9% (95% CI 88.0–96.3%), o3 95.9% (91.7–98.3%), Copilot-Quick 93.5% (88.7–96.7%), Copilot-Deep 94.1% (89.4–97.1%), Gemini-Flash 86.5% (80.4–91.2%), and Gemini-Pro 95.3% (90.9–97.9%). Post-hoc testing indicated that Gemini-Flash was significantly less consistent than o3 (*P* = 0.033) and Gemini-Pro (*P* = 0.035); all other comparisons lost significance after Benjamini–Hochberg adjustment.

### Response latency

**Fig. 4:**
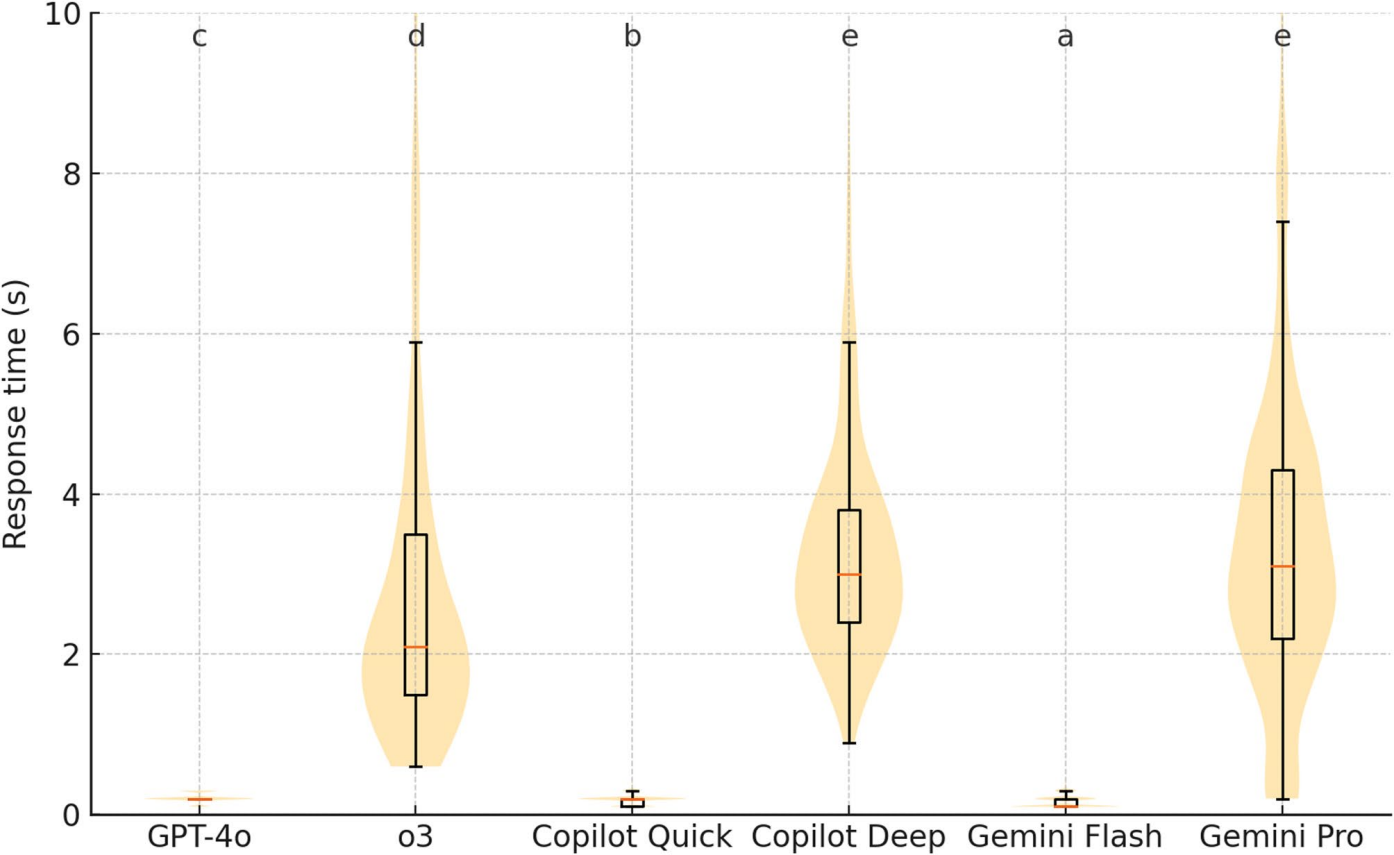
Violin-and-box plot of response-time distributions for all six models. Labels that share an identical lowercase letter (**a-e**) indicate groups with no significant Mann–Whitney U difference after multiplicity adjustment.

Median response times differed markedly across engines (Kruskal–Wallis *H* = 8092.0, *P* < 0.001; Table 2; Fig. 4). The lowest median came from Gemini-Flash (0.1 s, IQR 0.1–0.2 s), followed by Copilot-Quick (0.2 s, 0.1–0.2 s) and GPT-4o (0.2 s, 0.2–0.2 s). Among the deep-reasoning models, o3 (2.1 s, 1.5–3.5 s), Copilot-Deep (3.0 s, 2.4–3.8 s), and Gemini-Pro (3.1 s, 2.2–4.3 s) were considerably slower. Pairwise Mann-Whitney U tests with Bonferroni correction showed every comparison significant (*P* < 0.05) except Copilot-Deep versus Gemini-Pro (*P* = 0.717). Within-model analyses revealed that GPT-o3, Copilot-Deep, Gemini-Flash, and Gemini-Pro produced significantly faster responses on items they answered correctly, whereas GPT-4o and Copilot-Quick displayed no timing difference between correct and incorrect outputs.

**Table 2:** Median response times for correct versus incorrect answers across Language models. Results are presented as the median response time with interquartile range and full range for each of the six Language models. Correct-versus-incorrect timings were compared within models using Mann–Whitney U tests, and the resulting P-values were adjusted by the Benjamini–Hochberg procedure.

Model	False answer	True answer	Total	*P* value
GPT-4o	0.2 (0.2–0.2) 0.1–0.3	0.2 (0.2–0.2) 0.1–0.3	0.2 (0.2–0.2) 0.1–0.3	0.526
GPT-o3	2.5 (1.8–4.5) 0.7–15.5	2.0 (1.5–3.4) 0.6–15.5	2.1 (1.5–3.5) 0.6–15.5	< 0.001
Copilot Quick	0.2 (0.1–0.2) 0.1–0.4	0.2 (0.1–0.2) 0.1–0.4	0.2 (0.1–0.2) 0.1–0.4	0.066
Copilot Deep	3.6 (2.8–4.3) 0.9–8.4	3.0 (2.3–3.8) 0.9–21.0	3.0 (2.4–3.8) 0.9–21.0	< 0.001
Gemini Flash	0.2 (0.1–0.2) 0.1–0.3	0.1 (0.1–0.2) 0.1–0.4	0.1 (0.1–0.2) 0.1–0.4	0.013
Gemini Pro	3.5 (2.5–4.6) 0.2–12.6	3.0 (2.2–4.3) 0.2–12.6	3.1 (2.2–4.3) 0.2–12.6	0.022

## Discussion

This prospective in-silico diagnostic-accuracy study demonstrates that reasoning-optimised LLMs outperform their latency-optimised counterparts on a comprehensive bank of oral-maxillofacial surgery MCQs. In line with our primary hypothesis, the deep-reasoning engines, including OpenAI o3, Microsoft Copilot-Deep, and Google Gemini-Pro, achieved significantly higher overall accuracies than GPT-4o, Copilot-Quick, and Gemini-Flash, respectively (absolute gains of 5.9, 3.8 and 6.2% points), and an omnibus χ² test confirmed a meaningful performance gap across all six engines. Accordingly, we accept the alternative hypothesis that enhanced reasoning depth translates into superior answer selection. However, the expectation that this advantage would be uniform across all content areas was only partially met, in which significant differences emerged in five of twelve domains (anatomy, craniofacial deformity, cosmetic procedures, orthognathic surgery and sleep apnoea, and trauma), prompting rejection of the subsidiary hypothesis of domain-independent benefit. Finally, the markedly longer median response times recorded for o3, Copilot-Deep, and Gemini-Pro relative to their speed-tuned counterparts confirm our ancillary prediction of a speed–accuracy trade-off, further supporting acceptance of this secondary hypothesis.

From an educational and clinical-decision-making perspective, a 3–6%-point uplift in answer accuracy is far from trivial. On a 100-item board-style examination, it equates to three to six additional correct responses, which could be the difference between a borderline “pass” and “fail” for undergraduate or specialist trainees, and therefore a tangible safeguard against knowledge gaps that could propagate into patient care. Clinically, every incremental improvement lowers the likelihood that an algorithmically generated question misguides learners about critical protocols, where even a single misconception can have serious consequences. Moreover, cumulative gains across an entire curriculum mean that learners who rely on reasoning-optimised LLMs answer dozens more questions correctly over a semester, consolidating deep conceptual frameworks rather than superficial pattern recognition. In short, what appears numerically modest at the aggregate level translates into a meaningful enhancement of learner safety, assessment validity, and ultimately patient outcomes.

Domain-level scrutiny showed that the performance differential between the reasoning-optimised and latency-optimised engines was concentrated in seven of the twelve content areas, most notably trauma, craniofacial deformity, and orthognathic surgery. Questions in these domains typically embed multilayer clinical vignettes that demand sequential spatial reasoning (e.g., mapping fracture patterns to fixation order), numeric or algorithmic calculations (e.g., cephalometric metrics), or the integration of several modifiers (age, comorbidity, fracture line). Such “multi-hop” cognitive load aligns with architectures that generate longer chains of thought and exploit larger context windows, giving the reasoning-optimised model a tangible edge. By contrast, domains with negligible gaps are dominated by single-step factual recall that both engines retrieve with similar efficiency in a single sampling pass. Hence, the observed domain effect is best explained by the depth of reasoning required, not by content volume per se. Whenever an item can be solved through a solitary memory lookup, the speed-tuned model closes the gap, but when success hinges on chaining three or more inferential steps, the deeper architecture maintains its superiority.

Several contemporary evaluations of LLMs in dentistry provide a useful backdrop for our findings. On a 714-item oral and maxillofacial surgery board dataset, Mahmoud et al.^9^ reported GPT-4o as top performer at 83.7% accuracy, markedly ahead of Gemini (66.9%) and Copilot (62.2%). Quah et al.^10^ confirmed a similar hierarchy on 259 university-level oral and maxillofacial surgery questions, with GPT-4 reaching 76.8% and Copilot 72.6%. Outside surgery, Eraslan et al.^11^ showed Copilot leading a prosthodontic DSRE cohort at 73%, with Gemini 63.5% and Claude 57.9%, while Ramlogan et al.^12^ observed GPT-4 and GPT-4o sustaining 77–78% on a final-year periodontology exam, about 17 points above student means. Conversely, Jeong et al.^13^ found that no chatbot exceeded 65% on an oral-maxillofacial radiology test, underperforming relative to students. Against this landscape, our deep-reasoning Gemini-Pro (88.3%) and OpenAI o3 (87.3%) set a new accuracy ceiling for dental education, outperforming the best prior oral and maxillofacial surgery result by 4–5% points and eclipsing non-surgical benchmarks by 10–25 points. Crucially, none of the earlier studies quantified inference latency or contrasted “reasoning-optimised” with “speed-optimised” variants from the same vendor. By pairing accuracy with batch-level response times, we provide the first evidence in oral and maxillofacial medicine that gaining an extra 3 to 10 correct answers per 100 items costs roughly 2 to 3 s in response time. This empirical guidance is absent from investigations that report only a single metric and is directly relevant to educators who must balance precision against real-time usability.

Mechanistically, reasoning-optimised engines outperform their speed-tuned siblings because they sustain a deeper internal chain of thought, executing more inference steps before emitting the first token, and expose markedly larger context windows that let them juggle multi-layer clinical vignettes without truncation^8,14^. Our latency analysis further showed that, in these deep models, items ultimately answered incorrectly took approximately 0.5 s longer than correct items, implying that when early reasoning fails the model extends its thought chain in search of a viable path; speed-oriented variants, by contrast, truncate this search and therefore display little timing difference between right and wrong answers. Educationally, this trade-off argues for a tiered deployment: reasoning-optimised models (o3, Gemini-Pro) should act as high fidelity item writer assistants when curating summative question banks, while latency-optimised counterparts (GPT-4o, Copilot-Quick, Gemini-Flash) suit real-time objective structured clinical examination simulations and rapid-fire quizzes where sub-second turnaround preserves learner flow. In both cases, coupling the chosen LLM with faculty oversight remains essential. Expert review curbs hallucinations, calibrates difficulty, and adds pedagogical context, whereas the model accelerates coverage and flags content gaps, yielding a hybrid workflow that maximises reliability without forfeiting the efficiency gains demonstrated here.

Although reasoning-optimised engines lifted headline accuracy, 73 items (≈ 3%) still defeated every model, exposing three consistent failure modes. First, ultra-rare single-value facts (e.g., 80% long-term fat-graft loss, 60% ketamine hallucinations) are sparsely represented in pre-training data. Second, micro-anatomic minutiae, such as the nerve endangered in iliac-crest graft harvest and the artery feeding the nasal tip, demand detail that even specialist texts mention only briefly. Third, negatively worded or “except” stems invert the required logic; models’ chains of thought often derail at this final Boolean flip. These weaknesses cluster in domains packed with numeric thresholds and fine landmarks. The orthognathic surgery and sleep apnea domain shows the lowest accuracy (68%), whereas the infection and pathology domain, rich in guideline phrases, peaks at 92%. The pattern implies that LLMs struggle when answers hinge on rare facts or negations rather than broadly distributed biomedical knowledge, underscoring the need for retrieval-augmented prompts and mandatory human vetting of such items.

This prospective, six-engine comparison draws strength from its use of a large, standardised MCQ bank and domain-stratified statistics, providing the first multi-faceted view of speed-accuracy trade-offs across the OMFS curriculum. Yet the work remains in silico because it measures neither learning gains nor item writing quality in real learners, relies on a single English language question source and fixes the prompt to a minimal template, thereby sacrificing ecological realism for experimental control. Possible biases include inadvertent exposure of test items in model training corpora, “hidden” chain-of-thought leakage via API logs, and the absence of an independent item-difficulty calibration that could clarify whether failures reflect model limits or question complexity. Our scope was intentionally limited to single-best-answer MCQs to ensure commensurable accuracy metrics and fair latency comparisons. Future work will therefore preregister a multi-format evaluation (multi-select and open-ended) with explicit scoring rules and item-response modelling on larger, multi-institutional repositories to test whether the observed speed–accuracy relationship generalises across item formats. In parallel, a multilingual, multi-institution randomised classroom trial will assess whether these architectural choices improve students’ knowledge retention, assessment validity, and patient-care-relevant outcomes. These constraints notwithstanding, the findings position deep-reasoning LLMs as high-fidelity assistants for standardised item banks, while latency-optimised variants suit real-time simulation.

## Conclusions

In summary, reasoning optimised large language models deliver a clear accuracy advantage over their latency-tuned counterparts on complex oral and maxillofacial surgery MCQs, confirming that deeper chains of thought translate into more reliable answer selection. This gain, however, is accompanied by a two to three second response-time penalty and residual blind spots in rare fact recall and negated stems. Broad deployment should therefore be preceded by a quality-assurance workflow that pairs high-fidelity engines with expert review and calibrated item pools, ensuring that the benefits of deeper reasoning are realised without compromising efficiency or educational integrity.

## Supplementary Information

Below is the link to the electronic supplementary material.

Supplementary Material 1

## Data Availability

All data generated or analyzed during this study are included in this published article and its Supplementary Information file (Dataset.xlsx).
